# Cervical cancer screening barriers and facilitators from the perspectives of women with a history of criminal-legal system involvement and substance use

**DOI:** 10.1186/s40352-024-00262-z

**Published:** 2024-02-26

**Authors:** Amanda Emerson, Marissa Dogan, Elizabeth Hawes, Kiana Wilson, Sofía Mildrum Chana, Patricia J. Kelly, Megan Comfort, Megha Ramaswamy

**Affiliations:** 1 KU School of Nursing, University of Kansas Medical Center, 3901 Rainbow Blvd, Kansas City, KS 66180 USA; 2 Department of Psychiatry and Behavioral Neurobiology, University of Alabama at Birmingham-Heersink School of Medicine, 530 Beacon Parkway West (Suite 701), Birmingham, AL 35209 USA; 3 Jefferson College of Nursing, Thomas Jefferson University, 901 Walnut Street, Philadelphia, PA 19107 USA; 4 RTI International, San Francisco, CA USA; 5 Department of Population Health, University of Kansas Medical Center, 3901 Rainbow Blvd, Kansas City, KS 66106 USA

**Keywords:** Cervical cancer, Papanicolaou test, Access to care, Screening, Prison/jail, Vulnerable populations

## Abstract

**Background:**

The wide availability of routine screening with Papanicolaou (Pap) tests and vaccinations against *human papillomavirus* has resulted in a decline in rates of cervical cancer. As with other diseases, however, disparities in incidence and mortality persist. Cervical cancer, is found more often, at later stages, and has worse outcomes in people who live in rural areas, identify as Black or Hispanic, and in people who are incarcerated. Studies report 4–5 times higher rates of cervical cancer incidence in people detained in jails and prisons than in community-based samples. Studies to explain cervical cancer differences have been inconclusive, though there is broad consensus that issues of access play a role. In this study, we sought to learn more from people who have a history of criminal-legal system involvement and substance use about what barriers and facilitators they perceive in accessing cervical cancer preventive health and other support services in the community.

**Results:**

We conducted semi-structured interviews with open-ended questions by telephone with 20 self-identified women, ages 22–58, in Birmingham, Alabama. Interviews were audio recorded and transcribed and the transcripts analyzed using immersion-crystallization techniques. Our team identified two main themes, *making connections: the importance of interpersonal communication*, which stressed barriers and facilitators related to what makes for effective and humanistic interactions in cervical health prevention and other services, and *getting it done: the logistics of access and availability*, which highlighted elements of cost and payment; scheduling; transportation; and clinic policies.

**Conclusions:**

People with a history of criminal-legal system involvement and substance abuse meet with a variety of enabling and impeding factors at personal and interpersonal as well as systemic levels in obtaining cervical health services. To better ensure that women in this high-risk group have equitable access to cervical cancer prevention and treatment—and thus better cancer outcomes—will require multilevel efforts that include an emphasis on improving the human connection in health care encounters and improving the nuts-and-bolts logistics related to accessing that care.

## Background

Over 4,000 people die every year in the United States from cervical cancer. Epidemiologically, the cause of cervical cancer is straightforward; malignant neoplasms of the cervix are almost always triggered by a long-term infection with a high-risk strain of *human papillomavirus* (HPV). HPV is a common sexually transmitted infection that spreads through skin-to-skin contact and only rarely develops into cancer. Cervical cancer can be controlled effectively through primary prevention via vaccination or through secondary and tertiary prevention through regular screening, follow-up, and treatment. Over the past 30 years, secondary prevention by routine screening with the Papanicolaou (Pap) and more recent HPV testing has had an enormous impact on rates of cervical cancer (National Cancer Institute, 2022). In the 10 years after introduction of HPV vaccination in 2006, which initially focused on young people ages 9–15, cervical cancer rates fell 78% in the high-incidence 20-24-year-old group (Markowitz et al., 2019). Even while prevention strategies have drastically reduced the prevalence of cervical cancer, not all groups have benefitted equally.

Differences in rates and outcomes of cervical cancer have been attributed to unequal access to services; differences in HPV and cervical cancer awareness, knowledge, and perceived susceptibility; and beliefs related to vaccination (Zeno et al., 2022; Pratte et al., 2018; Blake et al., 2015). Relevant to the present study, women with a history of criminal-legal system (CLS) involvement have disproportionately high rates of abnormal cytology and face multiple challenges in receiving cervical screening services and following-up on abnormal results (Kelly et al., 2017; Jodry et al., 2021). Our purpose in this study was to understand, from women’s own perspectives, how barriers and facilitators are experienced by women in accessing cervical cancer preventive and allied health services in the community.

When it comes to cervical cancer, factors like rurality, geography, and race play a role in who develops cervical cancer and what outcomes they have (Cohen et al., 2023). In analysis of over 80,000 cervical cancer cases diagnosed in the U.S. between 2000 and 2018, Cohen et al. (2023) found Black people with either of two histological subtypes of cervical cancer (squamous cell or adenocarcinoma), staged as either regional or distant (i.e., metastatic), had “dramatically” lower 5-year survival rates and shorter survival times than White or Hispanic people. Mortality from cervical cancer occurs nationally at a rate of 3.4 per 100,000 Black people and 2.0 per 100,000 White people (American Cancer Society, n. d.). Cervical cancer disparities are likely a product of gaps along the cancer control continuum—vaccination, screening, follow-up, and treatment. Unequal outcomes reflect deep-seated inequities around race that manifest in how providers and systems preferentially inform, recommend, charge for, and otherwise facilitate cervical care in White versus Black people (Ford et al., 2021).

Where one lives has an impact. Incidence rates tend to be higher in rural than urban areas and in the South, Midwest, and Western Plains states than those in the Northeast, North Central, Southwest, and Pacific Coast (U.S. Centers for Disease Prevention and Control, 2018). Prevention and follow-up care can be more difficult in rural areas, due to barriers of availability, cost, transportation, and time. Geographic disparities are prominent in the largely rural state of Alabama, where this study focuses. Overall, Alabama’s cervical cancer incidence in 2010–2019 was 9.1 per 100,000 women, significantly higher than the U.S. rate of 7.5 per 100,000. Incidence in Alabama’s 55 rural counties was significantly higher (9.6 per 100,000) than in its 12 urban counties (7.7 per 100,000) (Alabama Public Health, 2022).

Cervical cancer disparities reflect complex structural vulnerabilities that flow from systemic inequities in access to health and to health services by race and socioeconomic status. These vulnerabilities intersect in those who have CLS involvement. Researchers comparing annual pooled data from U.S. jails and prisons with community-based Behavioral Risk Factor Surveillance System (BRFSS) data found much higher rates of cervical cancer in jails (OR 4.16; 95% CI 3.13 to 5.53) and prisons (OR 4.82; CI 3.74 to 6.22) than in the general population (Binswanger et al., 2009). Racism, too, is embedded in the system of mass incarceration (in patterns of arrest, charging, prosecution, and sentencing), with Black women almost twice as likely as White women to serve time in a jail or prison (Alexander & West, 2012; Sentencing Project, 2022). Drug laws have fallen particularly harshly on women in general, many of whom *get* to incarceration along a path that winds from violence and abuse to trauma disorder to substance use (Gehring, 2019; Kelly et al., 2014; Sawyer, 2019). Importantly, incarceration rarely lasts a lifetime: most people are released and reenter the community in less than a month (Sawyer, 2019), though many do so repeatedly. In 2016, more than 41,000 women were released from Alabama jails and prisons (Sawyer, 2019), many returning to the challenging life circumstances that preceded incarceration, except now with the added stigma of a criminal record and further hurdles that that history poses to employment, housing, and health care.

It is well understood that on their own and in combination incarceration and substance use can interfere with health, impeding cancer risk reduction and preventive services use (Salyer, Lipnicky et al., 2021). What is far less clear is what that interference (or its absence) looks like from the point of view of the women who experience it. We conducted this study to better understand how women with a history of CLS involvement and substance abuse perceived experiences of seeking and accessing cervical cancer preventive and allied services so that we might be more successful in designing and delivering care that meets their needs.

## Methods

This was a qualitative descriptive study of data from one-on-one, semi-structured telephone interviews with women who have a history of CLS involvement and substance abuse. The data were collected as part of the *Tri-City Cervical Cancer Prevention Study* (2019–2024), a mixed methods natural history study. *Tri-City* also included annual surveys over three years to compare women’s access to and use of cervical health and supportive services of women with a history of CLS involvement in three U.S. cities with diverse health funding and resource environments: Birmingham, Alabama; Oakland, California; and Kansas City, Kansas/Missouri (Salyer, Lee et al., 2021). Institutional Review Board approval (#142054) was granted by the University of Kansas Medical Center, with reliance agreements in Birmingham and Oakland.

### Sample

Participants in *Tri-City* self-identified as women, were 18 years of age or older, and had a history of incarceration or other CLS involvement (probation, parole, court-ordered residential treatment). The total baseline survey sample in *Tri-City* was 510 participants, with 20 participants sampled for interviewing from the survey group in each city. In this analysis, we elected to focus in-depth on the Birmingham interview cohort (*n* = 20) because Alabama offers a particularly stark example of inequity, with the fourth highest rate of cervical cancer in the US, reflecting 10.1 new cancers per 100,000 women in 2019 (U.S. Cancer Statistics Working Group et al., 2021). Alabama has also had steep 40-year rate increases in women’s incarceration—711% in jails and 583% in prisons (Vera Institute, 2019). All the women in the Birmingham study cohort were affiliated with an outpatient community corrections facility that provides court-ordered monitoring and case management services. Additional details about the overall *Tri-City* sample, the constituent pre-existing study cohorts, and sampling procedures can be found in (Salyer, Lee et al., 2021). Participants who interviewed gave informed consent (documentation waived) prior to participation in as part of the *Tri-City* consent process. All who completed an interview were thanked for their time with a gift card.

### Data collection

A research team member trained in trauma-informed interviewing administered the interviews via telephone between February and September 2021. Our semi-structured guide of 15 items (Table 1) was based on the aims of *Tri-City* and our previous research and was reviewed and revised by a panel of women’s health physicians who work with women who have CLS involvement and socioeconomic vulnerability. Interviews were roughly 30 to 60 min in length.

**Table 1:** Interview guide

Stem Question	Follow-ups
1. I’d like to begin by hearing about a time when you needed a specific health care service and you had a positive experience. This could be a time when you went for health care of any kind, mental health services, help with substance use, help with the court system, housing services, or other services. Talk me through what you did and what happened.	• What did you need help with? • How did you go about getting help? • Where did you go? • What steps did you take? • What people do you remember talking with or interacting with? • How were you treated? • What were specific problems or barriers you ran into? • How did the experience end? • What made the experience a positive one?
2. Now can you tell me about a time when you needed a specific health care or other kind of service, but you could not get it for some reason or things did not go well. Please talk me through what happened.	• What did you need help with? • How did you go about getting help? • Where did you go? • What steps did you take? • What people do you remember talking with or interacting with? • How were you treated? • What were problems or barriers you ran into? How did the experience end? • What made the experience a negative one?
3. Can you tell me about the kinds of places you go for routine sexual health care, such as Pap tests, STD tests, and birth control. How did you end up going to these places to get care? What kinds of health care do you get at each?	• Probe for Pap tests, STD tests, and birth control; if any are not obtained at the stated place, ask: Where do you go when you need a Pap test/STD test/birth control? • How did you find this place to get care? • How do get there? • How you pay for care at the places where you go?
4. Thinking back to your last Pap test, what made you decide to go for the Pap test?	• How did you get to the place where you got the Pap test? • How did you pay for the test or get health insurance to cover it?
5. What do you remember about the health care provider who gave you your last Pap test? Was it a doctor, nurse practitioner, midwife, or a physician’s assistant? What were your interactions with that person like?	• What, if anything, did the health care provider do that helped make you feel more comfortable? • What, if anything, did the health care provider do that made you feel uncomfortable?
6. Can you describe the Pap test itself—what happened during the Pap test?	• Do you remember how the health care provider explained the reason for the Pap test? • What did they say it was for? • Were you able to ask questions? • Did you see the speculum? • What other instruments were used? • Do you remember if the provider mentioned “HPV testing”? • Did they say they were going to send cells to be analyzed for HPV types?
7. What did the health care provider say about how they would get your results to you? Can you remember how you actually ended up getting the results?	• What were the results? • What did you do after getting the results? • Did you follow-up?
8. What would you change about the experience of your last Pap test?	
9. Sometimes women might need a Pap test, but they find it hard to get one. What are some of the things a person needs in order to get a Pap test?	• Ask about transportation, appointment scheduling/reminders, time off work, child care, health insurance, cash for co-payments, access to free or low-cost clinics; for each.
10. What do you remember about the last time you thought you needed to get a Pap test but you *didn’t* get one?	• Why did you think that you needed to get a Pap test? • What were the things that got in the way of going to get the test?
11. What kinds of people in the community have helped you get Pap tests or other types of health care?	• Tell me about a time that you went for a Pap test and someone else encouraged you or helped you make that happen.
12. Tell me about a time when someone you knew or someone you didn’t know, like someone at an office or agency, got in the way of your getting a Pap test or other health care? What happened?	
13. If you have ever been in jail or prison, did you get a Pap test while you were there? If so, can you tell me about the last time you got a Pap test in those places – what was it like? Walk me through what happened.	• Why did you get it? • What were the health care providers like? • How did the health care provider explain why you needed the Pap test or what the results were? • Do you have anything to say about how that care compares to care you usually get in the community
14. If you do use or have used drugs or alcohol in the past, was there ever a time when drug or alcohol use got in the way of your getting health care? Again, I’m especially interested in Pap tests, but any kind of health care is fine, too.	
15. What advice would you give to a woman who wanted to get a Pap test? What would you tell her to do?	• What advice would you give to a woman who hasn’t had a Pap test and doesn’t want to get one?

### Data analysis

The interviewer audio recorded the interviews which were then transcribed by a professional service and reviewed by a team member for accuracy. The first author assigned pseudonyms. The first five authors analyzed the interview transcripts in stages, roughly following an immersion-crystallization process (Borkan, 2022). Immersion-crystallization is a process of organization, interpretation, and corroboration of meanings in a set of data. The approach requires substantial time for iterative critical reflection (Crabtree & Miller, 2023). The authors first immersed themselves in the interview transcripts, independently open-coding transcripts and then meeting as a group to discuss impressions. The group was well-prepared for this step by the training in trauma-informed and story-based interviewing that study team members completed at the beginning of the parent study that focused on recognizing the impacts of experience and standpoint on both interaction and interpretation. Second, the authors divided up the most prominent codes and wrote analytic memos in which patterns connected to the study goals and previous research were tracked across transcripts and interpreted as themes (i.e., crystallized). The thematic memos were discussed as a group and, in a final step, the first two authors returned to the transcripts to reexamine for overlooked and divergent elements and select passages for exemplification. The first five authors met twice-monthly over 14 months to analyze data and draft and revise the report. The sixth, seventh, and eighth authors provided corroboration through critical readings and the sixth helped shape the manuscript. We promoted rigor by using a systematic, iterative approach to analysis and multiple researchers.

## Results

### Participant characteristics

The 20 participants in this study were aged 22–58 years and included nine women who identified as Black and 11 who identified as White. None of the women identified as ethnically Hispanic or Latina. In the *Tri-City* baseline surveys, these participants reported experiences with incarceration that ranged from a few days in a county jail to 16 years in a federal prison. Nearly all reported current or past use of illicit substances, with 18 acknowledging having felt “bad or guilty” about their substance use, and eight reporting a drug overdose. Over a third of the women indicated that they had been treated unfairly by health care providers because of their history of criminal-legal system involvement and/or their substance use. In the qualitative findings below, we identify thematic patterns that help elaborate and provide insight into the women’s experiences.

### Themes

The interviews addressed positive and negative health and social services experiences, with emphasis on barriers and facilitators of cervical cancer prevention, both secondary and tertiary. We identified themes that clustered in two groups: (a) *making connections*, or the importance of interpersonal communication, and (b) *getting it done*, or the logistics of access (Table 2).

**Table 2:** Themes and subthemes with examples

Sunthemes	Participants	Examples
**Theme: Making Connections: Interpersonal Communication**		
***Quality: Providers who do or do not***		
• Take time, listen	Desiree, Jaelyn, Praise, Qatya, Raleigh	*I mean, she talked to me like, and explained things to me […] where I could understand it and I knew exactly what they were going to do, like why they were doing like whatever swabs they were doing and stuff like that. She actually just talked to me … like about, asking if I have kids and how old they were, asking me about my personal life. (Mimi)*
• Share information, involve patient in care	Cassie, Evan, Mimi, Isidore, Jaelyn, Lindsey, Raleigh	
• Provide person-centered, non-judgmental care	Cassie, Isidore, Jaelyn, Lindsey, Mimi, Qatya, Seylon	
**Theme: Getting it Done: Logistics of Access & Availiability**		
***Scheduling***		
• Phone queues, months’-out scheduling	Cassie, Desiree, Evan, Lindsey	*It’s not good because you have to be up at 7:15 and you, you have to wait on the phone until they count down the numbers, and most of the time, by the time they got to me all the appointments was full for the day. (Desiree)* *One time I didn’t get one [Pap exam], I just started a job and it was within my 90 days so I didn’t get it. (April)* *They give you a piece of paper, tell you you got to go here, got to go there. I had the hardest time, when I was homeless, getting my teeth pulled. Like they had me going here, had me going there. […] I can pretty much sum it up like this: things that other people do, I cannot do. (Evan)* *I got my blood work, I got a call from the doctor yesterday saying [my liver enzyme] levels were high […] And I can’t get in to [see] a doctor because I don’t have insurance. I tried calling the Health Department, they gave me the hospital […] and they want me to pay out of pocket, and I don’t have a job. (Praise)* *I have a lot of back pain and I have polycystic fibrosis and all kinds of other issues. I have to go through a lot of pain. So […] they just kind of thought I was coming in and seeking drugs because I was talking about how bad my pain was and looking at how many times I have had to go to the ER. (Raleigh)*
• Overscheduling, cancelling, waits	April, Cassie, Evan, Farah, Lindsey, Seylon	
***Getting away-Getting there***		
• Getting time off/missing work	April, Farah, Gemini, Jaelyn, Nan, Pamila,	
• Arranging child care	Cassie, Jaelyn, Pamila, Desiree	
• Arranging/accessing transportation	Bella, Cassie, Desiree, Farah, Jaelyn, Kay, Mimi	
***Payment***		
• Cost of/paying for treatment	April, Bella, Farah, India, Jaelyn, Gemini, Nan, Praise, Raleigh	
• Managing/avoiding medical debt	Jaelyn, Raleigh	
***Clinic policies & practices***		
• Pain treatment policies	April, Bella, Isidore, Mimi, Seylon	
• Chart bias practices	Cassie, Evan, India, Jaelyn, Farah, Lindsey, Pamila, Praise, Raleigh	

#### Making connections: the importance of interpersonal communication

When asked to describe positive and negative cervical and other health care experiences, the women’s responses often pointed to informative, empathetic, and nonjudgmental communication as a key positive element. Participants valued the time health care providers took to explain procedures and the care with which they answered questions. These simple acts allowed participants to feel comfortable, validated, and at ease. The women seemed particularly appreciative of providers who gratified their need for information, by inviting questions, giving and explaining options, and including the women in a process of inquiry or problem-solving. Cassie, age 23, and Bella, age 37, recalled providers who helpfully “broke [things] down,” and Desiree, age 33, and Lindsey, age 51, both said their providers wrote out lists of what was done during their visits. Jaelyn, age 47, recounted a diagnostic loop electrosurgical excision procedure (LEEP) procedure to remove cells on her ovary, during which providers “help[ed] me learn things about my body.” April, age 30, praised her gynecologist for answering her questions and engaging her directly by asking “did you understand?” and “did I answer your question?”

Person-centered, non-judgmental communication that acknowledged women’s humanity and met their need for comfort often made up for other more negative aspects of care. Mimi, age 37, described a Pap exam during an incarceration that was conducted in what appeared to be a storage room or “broom closet.” The participant recalled random machinery and an air conditioner secured in a window with duct tape, “not sterile at all.” In spite of the inauspicious surroundings, Mimi found the exam procedure a comparatively positive experience because the nurse asked about her kids and “actually just talked to me.” Isidore, age 55, on learning she needed additional testing and a biopsy following her Pap exam, was encouraged by her physician’s assuring her that they “would do whatever was necessary to make sure everything was okay.” She recalled an “extremely uncomfortable” cervical biopsy that was made less stressful by the provider’s continually telling her that she was “doing fine” during the procedure. Jaelyn and Lindsey, both patients at a local low-cost, faith-based clinic, specified that providers made them feel comfortable by praying with them at the beginning of their cervical cancer screening appointments.

Conversely, other participants reported a lack of warmth or acknowledgment of their feelings, even in some cases, their humanity. Seylon, age 46, recalled the uniform brusqueness of one gynecologist whom she remembered saying little during a Pap exam other than, “Okay, scoot to the end of the table.” Lindsey, age 51, described a dilation and cutterage (D&C) performed after she suffered a precipitous miscarriage in the bathroom at home. Lindsey’s own provider was unavailable for the procedure, and the provider who performed the D&C made her feel “like a piece of meat” and “just another number.” No one acknowledged Lindsey’s feelings: “My child was in the toilet. It wasn’t even considered that something very horrible had just happened.” Cassie observed that some providers, who seem always to be in a rush, “just stick [the speculum] in, open it up […], don’t really take into knowledge that, ‘Hey, I am working with a human here.’”.

Communication often took a negative turn when a participant’s substance use or incarceration became known to health care staff and providers. Mimi recalled how a nurse “doing my vitals looked at me like I was garbage because she saw my track marks. It was the worst feeling in the world. I left and didn’t go back.” Pamila, age 32, noted about substance use that health care professionals “don’t care or hear what you have to say, once they know.” Evan, age 29, similarly explained that once you “get something put by your name, people look at *that* and continue to judge you by that.” Farah, age 57, shared how, after she “slipped” and disclosed a past incarceration, her doctor’s “whole expression changed and she had a brand-new story,” subsequently altering Farah’s care plan and withdrawing a recommended procedure. Farah also described a series of cancelled Pap tests at one community clinic, wearily adding, “I hate to say it, but I think the prison was more willing to get [my] pap smear [done]. The appointments, for one, didn’t keep getting cancelled.” Most participants who described interactions with health care staff and providers while in jail or prison, however, characterized those experiences as alienating and dehumanizing. Qatya, age 54, summed up prison care with the two clear messages she received from nurses: “It’s a bother […] to see about you when you put in sick calls,” and “you’re just a low life with a number who can’t act right in society.”

The women we interviewed stressed the importance of positive communication in encounters with non-health-care social service workers who helped them identify and access resources. These communications often determined whether a participant was able to find and or access cervical care in the first place. Raleigh, age 30, put off health care because of the large medical debt she had accumulated, until one day a member of the staff took her aside and spent time explaining the hospital’s payment options “better and in a way that I could understand.” This allowed Raleigh to find a payment plan that she could manage. Henna, age 38, similarly described the help she received in figuring out a way to pay for a LEEP procedure. In other examples, participants described how workers in housing agencies, churches, and even jails and prisons passed along information that facilitated participants’ access to health and necessary affiliated resources. April, age 30, connected with a social worker at a hospital who introduced her to a program that provided bus passes, copay assistance, and information about preventive services. Farah, age 57, recalled how when she sought assistance from a sheltering and housing agency, the staff member she worked with “did everything she could to try to let me find somewhere to stay. I mean, she did *everything*. She stuck with me night after night just trying to help me.” Meaningful communication with a single person or a few staff members often made all the difference in a woman’s experience of health services.

Several participants described the facilitating role of friends and family who communicated information about providers they liked or referred free or low-cost clinics. Women found their women’s health providers through their mothers (Seylon) and aunts (Bella). They also saw in one another the potential for more than information and referrals. Women recognized the value in other women who could offer assurance and show that cervical screening is safe. Asked what she thought might help hesitant women get a Pap exam, Jaelyn, age 47, answered:

*I think the first thing they need is other women that they feel comfortable with, that they can talk* [with] *about personal things, so that they can become educated, and they can feel like, “Hey, when something’s going on with my body, I have someone that I can call [and ask] ‘What would you advise?’”* [They need someone who] *could get them to the doctor’s and know that they are going to be safe, that everything is going to go smooth.*

#### Getting it done: the logistics of access and availability

The second theme focused on how clinic and other facility or system practices facilitated or impeded women’s access to cervical and other health care. Especially prominent was the impact of *scheduling*. Cassie and Desiree described a same-day scheduling process at a local health department that required patients to call into a scheduling service at 7:15 a.m. where they were put in a queue and then waited on hold to be scheduled for the day’s appointments. Once the slots were filled, those still waiting were turned away for the day. Other women described “stacked up” appointments or overscheduling in which clinics made more appointments than they could accommodate, presumably thinking that some with appointments would not show. Crowded waiting rooms and long waits were common. Seylon, age 46, described how at one clinic the staff goes to lunch, leaving patients sitting in the waiting room until they’ve had their break.

The frustration of a long wait was compounded by the juggling necessary to do the waiting. Women described challenges of having to take off work for half a day, find childcare, and/or arrange for transportation. Jobs posed a barrier for some women. Four participants (April, Pamila, Farah, and Nan) described how their jobs made it difficult to schedule Pap exams, either because they were new employees or were reluctant or unable to ask for time off. As Pamila, age 32, said of her employer, “They’re like, ‘Oh, that’s something you take care of on your own time’—but you don’t *have* your own time.” Women with children struggled to schedule appointments around school or childcare. Cassie, age 23, who had to take her breastfeeding infant and two toddlers with her to a Pap exam, described trying to keep the children fed, entertained, and comforted throughout the wait and the exam—a task made more difficult because she was herself both anxious and uncomfortable.

Other women referred to difficulties finding, coordinating, or paying for transportation. Farah, age 57, described having to ride the bus to the bus station where you “sit there and wait. You don’t know whether it’s going to be an hour or two hours. You have to sit there and wait on the next bus. [Y]ou have to call and find out their schedules and stuff so you could try to be on time, but it’s really—sometimes the buses be late or sometimes they’ll show up early.” Kay, age 58, who accepts rides from her son, recalled when she stayed in a homeless shelter and regularly used the bus to get to her appointments despite physical pain riding the bus caused: “I done had so many broke bones,” she explained, “they [buses] don’t sit well with me.” Private arrangements came with their own pitfalls. Mimi, age 37, said her daughter had been late so often picking Mimi up for her appointments that Mimi began making up appointment times 30–45 min earlier than the actual ones to ensure she arrived on time. Another participant described a 90-minute round-trip drive to see her provider. A few of the women described being offered transportation voucher services or other ride assistance by health care clinics, homeless shelters, and community services programs. Cassie detailed a convoluted process of scheduling, requesting approval, and then waiting for a confirmation number for a ride service paid for by Medicaid.

A number of women identified payment or cost of health services as a barrier to health care, though not usually specific to cervical care. Only April and Raleigh, both age 30, said they had forgone routine cervical cancer screenings in the past due to lack of insurance, and Henna, age 38, referred to delaying a LEEP until she was informed of a payment plan. Participants also appreciatively described $5 office visits, sliding fees, and free Pap exams at local clinics. Several participants speculated that *other* women may have difficulty accessing cervical health services due to cost or lack of insurance. Farah, age 57, reminded us that even very low-cost options can be a barrier to some, observing, “sometimes things be so hard you don’t even have bus fare.” In general, health services for needs other than cervical cancer prevention tended to be more challenging. India, Praise, Gemini, and Nan, for example, described putting off surgery, specialist care for chronic seizures, follow-up for abnormal hepatic lab results, and treatment for depression due to lack of insurance. Nan, age 22, recounted the “domino effect” of not being eligible for Affordable Care Act, Medicaid, or Medicare, and not being able to get approved for disability because she could not pay to see an approved doctor to document her condition. Jaelyn, age 47, and Raleigh, age 30, both described avoiding or being refused health services as a result of medical debt.

Finally, clinic policies and practices around pain prescribing and mental health affected the participants’ health services access and use. The women stressed how personal histories of incarceration and substance disorder treatment, recorded in their medical records, influenced the treatment they were offered—and sometimes, as a result, how and what treatment they sought. Evan, age 29, described how, because of the alternative sentencing (“drug court”) in which she participated, she had to lie to medical providers about her substance use. India, age 45, recalled a scheduled follow-up appointment with a surgeon, in which, only two weeks into a new job and frustrated about having to miss work, she waited four hours in a waiting room and finally “ended up walking out.” The surgeon later called and denied her a pain medication refill, explaining that she “behaved like a drug addict.” Another participant described being turned away from a mental health facility following a suicide attempt because the facility did not accept actively using patients. Seylon, age 46, went to the emergency department for an abscess on her arm, where the provider asked,

“Is this from using drugs?” and I said yes, and I told him what I was using, because I wanted help, but the doctor wouldn’t give me anything—nothing but antibiotics—nothing for pain […]. Instead of saying, “Do you need any help?” or “You really need to get off it,” something like that, he didn’t say anything to me. He gave me antibiotics and said “Bye.” That was it.

Others described barriers to getting adequate pain management for injuries related to car accidents, dog bites, back pain, post-surgical pain, and pain associated with polycystic fibrosis—in each case, the women attributed providers’ unwillingness to prescribe pain medication to notations in their medical record about prior drug court involvement or substance use disorder and/or treatment. Such clinic- or facility-level practices, while not specific to cervical screening, were experienced by the women as discriminatory and stigmatizing, generally discouraging women from using the healthcare system. As one woman explained wearily, “Once you get put in the system and they put certain things on your name […], that’s all they will see.”

## Discussion

Cervical cancer is a largely preventable disease with incidence rates that have decreased dramatically over the past three decades. Even so, gains against cervical cancer lag behind goals in some groups and regions (Benavidez et al., 2021). Though likely due to COVID-19, rates of overdue screening have actually increased in recent years (Winstead, 2022). Women who are incarcerated report cervical cancer diagnosis at 4–5 times higher rates than community samples (Binswanger et al., 2009). CLS-involved women do not have higher rates of cancer *because* they spent time in jail or prison but because they share life circumstances and social determinants, including substance abuse, that put them on pathways more apt to end in incarceration, undetected HPV infections, and missed treatments. We focused this study on Birmingham, Alabama, which represents a challenging cervical cancer landscape for women who face marginalization due to overlapping influences of CLS involvement and substance use. Though racism was not explicitly referred to by the women we interviewed, we recognize that racial discrimination pervades systems of mass incarceration (Alexander & West, 2012); generational economic disadvantage (Chetty et al., 2020); and disparities of health, health coverage, and access to health services (Hill et al., 2022)—including women’s sexual and reproductive health services (Prather et al., 2018). Women shared their experiences of perceived barriers and facilitators to cervical cancer prevention in two primary clusters: *making connections* and *getting it done*.

The quality of patient-provider communication was one of the most common themes across interviews. Participants stressed provider empathy and engagement and put special emphasis on efforts made by providers to explain and inform. Studies of patient-centered care show that better provider-patient communications are associated with improved patient health, better patient experience, greater patient engagement in meeting health care goals, and in a few cases cost savings (Drossman et al., 2021; Grover et al., 2022; Haskard Zolnierek et al., 2009). In their systematic review and Delphi study, Drossman et al. (2021) recommended 10 provider-based approaches to enhance provider-patient communication, including understanding the patient’s agenda, validating, educating, reassuring, and being there. Women in our interviews related similar provider communication practices that influenced their willingness to seek cervical health services. It may be that women who are socially marginalized because of racism and/or CLS involvement require extra effort from health care professionals to build trust and communicate respect. Zulman et al. (2020) have recommended motivational interviewing and narrative medicine techniques, in which communication in health care encounters centers on a patient’s own story of their health, to enhance health care trust.

Similar to other modes of patient-centered communication, such practices likely benefit from training, and it is unclear whether health care professionals obtain adequate training in interpersonal skills, especially in communicating effectively with patients from racially or other minoritized groups who might experience medical distrust (Griffith et al., (2021). Communication and interpersonal skills were formerly included in the United States Medical Licensing Examination (USMLE) in the Step 2 Clinical Skills (CS) exam. Step 2 CS assessment used standardized patients to generate ratings of examinees’ communication and interpersonal skills: questioning skills, information-sharing skills, and professional manner and rapport (Winward et al., 2013). Unpopular among medical students and some faculty because of its cost, the Step 2 CS exam was suspended in 2021 to facilitate licensing during the COVID-19 pandemic and has not been resumed (Tsichlis et al., 2021). One implication of our findings is that there is more, not less need for training and testing of physicians (and nurses and other health care professionals) in communication and engagement skills, including those tailored to reach underserved patient communities and groups with diverse health literacy. Such training would offer focused instruction on how best to transmit cervical cancer prevention information—e.g., how providers explain the purpose, process, timeline, and outcomes of Pap and HPV testing and treatment. Indeed, previous study by our team indicates inadequacies in communication may explain some missed follow-ups after abnormal Pap results (Salyer, Lipnicky et al., 2021). In general, our interviews suggested that training needs to include emphasis on the manner or *how* information is conveyed, so women feel valued, heard, and included in their own care.

Women also described how social networks factored into cervical care through shared resources and information about providers, facilities, and the screening procedure itself. These often involved family and friends, but we also heard about the facilitation of care by front-line community workers. Research on community health workers, lay health advisors, and—in Hispanic communities—*promotoras* has demonstrated varied success in increasing cancer prevention care (Adams et al., 2021; Mboineki et al., 2021). At their best, such programs bridge cultural, communication, and confidence gaps between health professionals and patients by providing relatable, accessible education and navigation services (Luque et al., 2017). Community health worker and similar programs have been used to facilitate cervical cancer screenings in African American women living in rural Alabama (Mayfield-Johnson et al., 2016); Latina farmworkers in Texas (Mojica et al., 2016), and Korean American women living in the Baltimore-Washington, D.C. area (Han et al., 2017). Authors argue that community-based health education and navigation programs increase minoritized and underserved patients’ trust in what can seem like a detached, structurally biased, and overly complex healthcare system (Mayfield-Johnson et al., 2016).

Clinic practices was another area of challenge for women in obtaining cervical care. Appointment scheduling is a frequent topic in recommendations to improve primary health care but perhaps less often in cervical care (Irwin et al., 2015; Huang, 2016; Woodcock, 2022). The most recent Community Preventive Services Task Force (2018) recommendations for reducing structural cancer screening barriers included offering hours that better meet client needs, offering screening at alternative non-clinical sites, and easing administrative procedures of scheduling. But whereas these approaches were recommended to increase equity in colorectal and breast cancer screenings, the recommendation was withheld for cervical cancer since evidence of effects on cervical cancer screening rates was lacking. Along similar lines, Senkomago et al.’s (2021) comparison of screening barriers in women in a cancer registry and in a cancer survivors’ social network (*Cervivor*) found the barrier “Clinic hours were inconvenient” to be among the *least* common responses—though more common among the study’s racially diverse and socioeconomically disadvantaged social network cohort. Interestingly, in the same study, one of two most common facilitators of screening in the social network group was “Having screening tests with their annual exams,” suggesting that clinic hours might only partially capture the scheduling construct (Senkomago et al., 2021). Mkuu et al.’s (2022) study of clinicians’ views of cervical care barriers for women with behavioral health conditions (i.e., mental health and substance use) adds another perspective: in the clinicians’ view, the salient scheduling barrier was women’s “forgetting” to schedule appointments, not the availability or lack of ease in making appointments.

In our study, the safety-net clinics that the women used did not appear to offer much to meliorate scheduling barriers. In other contexts, facilities have used advanced access (or open) scheduling, in which primary care clinics, for instance, strive to offer all patients an appointment on the same day of their call, within 24 h of their call, or at a scheduled time of the patient’s choosing (Haggerty et al., 2018). Proponents of advanced access scheduling claim it improves wait times, health outcomes (since longer waits can mean cascading health issues), and patient satisfaction (Rose et al., 2011). In a systematic review of advanced access, Rose et al. observed that three of the five studies that focused on clinics serving patient populations with low socioeconomic status and included measurement of appointment no-shows, there were significant decreases in no-show rates after implementation of advanced access scheduling. When combined with women’s difficulties arranging transportation, childcare, and time off work, patient-averse clinic practices like inconvenient hours and challenging scheduling processes may decrease access to care in the short-term and increase cancer mortality in the long-term (Chatterjee et al., 2016). Payment mechanisms can also play a role. Senkomago et al.’s (2021) study of barriers and facilitators of cervical care identified full or partial insurance coverage among the most commonly noted enablers of cervical cancer screening. We know from previous research on barriers to follow-up after an abnormal Pap exam that women with past CLS involvement, in a state without Medicaid expansion, expressed hesitancy to schedule due to fears about cost (Kelly et al., 2017). For women *with* Medicaid, matters might not be much better. Hsiang et al. (2019) found that patients with Medicaid had a 1.6 lower likelihood of successfully scheduling a primary care appointment than patients with private insurance.

Experiences of discrimination impacted women’s use of cervical and other health services, especially in encounters in which physicians or nurses became aware of a participant’s history of substance use disorder treatment and/or CLS involvement. Researchers have documented health care providers’ adoption of an “avoidant approach” or detached attitude toward patients with a history of substance use disorder compared with other patients (Van Boekel et al., 2013), a disposition that was reflected in several women’s descriptions in the interviews. MacAfee’s (2020) study with women at substance use treatment facilities noted that stigma posed a significant barrier to women’s reproductive and sexual health care, including cervical cancer treatment and prevention. The participants in our study reported numerous incidents of stigmatizing behavior by health care workers arising from patient records documenting past incarceration and/or treatment for substance use. Such reports are concerning and indicate that more work needs to be done to ensure that patients who have life circumstances or social determinants that increase their vulnerability to social marginalization and health risk do not meet with further marginalization and increased risk because of providers’ bias and/or lack of interpersonal skills. Providers may need training in ways specifically to avoid stigmatizing communication in speech and body language. Zwick et al. (2020) identified steps providers can take to reduce stigma when providing care to those with substance use disorder: use of person-centered language, listening non-judgmentally, and treating all with dignity and respect. Zwick et al. recommended that providers also support measures to promote “equality and parity in medical coverage,” thus presumably challenging a merit-based logic of health care distribution. Another way providers might promote openness in their delivery of health services is through implicit bias training, initially through implicit association testing to recognize bias, and then through skills-building in equitable communication. As Cooper et al. (2022) have observed, skills to reduce implicit bias may best be developed when paired with mindfulness and emotional regulation training. The combination might be especially necessary in preventing stigma based on aspects of behavioral health like illicit drug use that are (problematically) attributed to patient choices or lifestyle and then harmfully legitimized as deserving of judgment (Dahl et al., 2022). As with many of the other recommendations implied by our findings, bias and stigma training would likely facilitate better access to services for women with CLS involvement across a range of conditions, not just cervical cancer prevention.

Limitations of our study included a convenience sample drawn from an existing study, whose participants’ experiences with cervical health and health care may not reflect those of the overall population of women with CLS involvement. Participants were also from a single large metropolitan area in a southern state that has historically struggled with low rates. Additional exploration is warranted to understand the cervical care experiences that may affect cervical cancer screening by women with CLS involvement who are Hispanic; who ascribe or are subject to belief systems that involve relatively greater constraints on who, when, and how they obtain reproductive health services; and among people who identify as sexual minority or non-binary.

## Conclusion

Health disparities in cervical cancer are patterned and persistent. They often reflect inequities in wealth and access as well as overt and covert biases against racial minorities and those with CLS-involvement and substance abuse histories. Barriers and facilitators to cervical cancer prevention occur both at the level of personal interactions and communications and in the nuts-and-bolts processes of learning about, scheduling, getting to, and paying for cervical cancer screening, follow-up, and treatment. Other health and social services encounters matter, too, in that they seemed to influence women’s attitudes about seeking preventive care at all. Our findings suggest that achieving cervical health equity for a high-risk group with CLS and substance use histories will require multilevel approaches to ensure that persons are appropriately trained and that systems and procedures are designed and functioning in ways that convey respect, support, and transparency in all encounters.

## Data Availability

The datasets used and/or analyzed during the current study are available from the corresponding author on reasonable request.
