# Correction: Cervical cancer screening barriers and facilitators from the perspectives of women with a history of criminal-legal system involvement and substance use

**DOI:** 10.1186/s40352-024-00295-4

**Published:** 2024-10-29

**Authors:** Amanda Emerson, Marissa Dogan, Elizabeth Hawes, Kiana Wilson, Sofía Mildrum Chana, Patricia J. Kelly, Megan Comfort, Megha Ramaswamy

**Affiliations:** 1 KU School of Nursing, University of Kansas Medical Center, 3901 Rainbow Blvd, Kansas City, KS 66180 USA; 2 Department of Psychiatry and Behavioral Neurobiology, University of Alabama at Birmingham-Heersink School of Medicine, 530 Beacon Parkway West (Suite 701), Birmingham, AL 35209 USA; 3 Jefferson College of Nursing, Thomas Jefferson University, 901 Walnut Street, Philadelphia, PA 19107 USA; 4 RTI International, San Francisco, CA USA; 5 Department of Population Health, University of Kansas Medical Center, 3901 Rainbow Blvd, Kansas City, KS 66106 USA

**Correction:** ***Health Justice 12,*** **9 (2024)**

10.1186/s40352-024-00262-z

Following publication of the original article (Emerson et al., 2024), corrections were made to three sources that remained anonymized in the published text, one of which also did not appear in the reference list. In Background, second paragraph, “Anonymized for Review, 2017; Anonymized for Review, 2021,” has been corrected to “Kelly et al., 2017; Jodry et al., 2021.” In Discussion, third paragraph, “Anonymized for Review, 2021,” has been corrected to “Salyer, Lipnicky et al., 2021.” The reference list has been updated to include: Jodry, D., Blemur, D., Nguyen, M. L., Kuhn, T., Easley, K., Wang, H., Ramaswamy, M., Birdsong, G., Kohut, A., Manobianco, B., & Flowers, L. (2021). Criminal justice involvement and abnormal cervical cancer screening results among women in an urban safety net hospital. *Journal of Lower Genital Tract Disease*,* 25*(2), 81–85. doi: 10.1097/LGT.0000000000000589.

The original article (Emerson et al., 2024) has been updated.
